# Controllable Coating Graphene Oxide and Silanes on Cu Particles as Dual Protection for Anticorrosion

**DOI:** 10.1021/acsami.3c08042

**Published:** 2023-08-07

**Authors:** Jinhua Sun, Kristoffer Harr Martinsen, Uta Klement, Alessandro Kovtun, Zhenyuan Xia, Plinio Fernandes Borges Silva, Eduard Hryha, Lars Nyborg, Vincenzo Palermo

**Affiliations:** † Department of Industrial and Materials Science, Chalmers University of Technology, Gothenburg SE-41296, Sweden; ‡ Institute of Organic Synthesis and Photoreactivity (ISOF), CNR, via Gobetti 101, Bologna 40129, Italy

**Keywords:** graphene, coating, Cu, anticorrosion, graphene-reinforced metal matrix composite

## Abstract

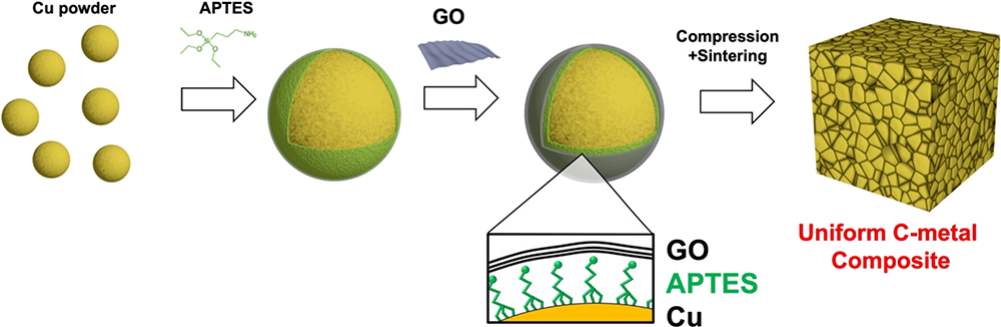

Although two-dimensional nanosheets like graphene could be ideal atomic coatings to prevent corrosion, it is still controversial whether they are actually effective due to the presence of parasitic effects such as galvanic corrosion. Here, we reported a reduced graphene oxide (RGO) coating strategy to protect sintered Cu metal powders from corrosion by addressing the common galvanic corrosion issue of graphene. A layer of silane molecules, namely, (3-aminopropyl)triethoxysilane (APTES), is deposited between the surface of Cu particles and the graphene oxide (GO), acting as a primer to enhance adhesion and as an insulating interlayer to prevent the direct contact of the Cu with conductive RGO, mitigating the galvanic corrosion. Due to this core–shell coating, the RGO uniformly distributes in the Cu matrix after sintering, avoiding aggregation of RGO, which takes place in conventional GO-Cu composites. The dual coating of GO and silane results in bulk samples with improved anticorrosion properties, as demonstrated by galvanostatic polarization tests using Tafel analysis. Our development not only provides an efficient synthesis method to controllably coat GO on the surface of Cu but also suggests an alternative strategy to avoid the galvanic corrosion effect of graphene to improve the anticorrosion performance of metal.

## Introduction

Corrosion, which causes damage to metal-based products and systems, is a major industrial problem. As estimated, the total cost of corrosion in the world reached about 2.5 trillion U.S. dollar.^1^ To counter this problem, various strategies of corrosion protection have been developed, for example, using polymers,^2^ oxide layers,^3^ and alloys^4^ as protective coatings on metal surfaces. In this regard, the coating materials play a crucial role in the anticorrosion performance and service life of the underlying metal. The widely used polymer-based anticorrosion coatings have to be relatively thick (in a millimeter scale) because the protective efficiency is proportional to the thickness.^2^ Moreover, a thick coating is required to lower the risk of leakage due to the presence of micropores formed during solvent evaporation.

Graphene, which is impermeable to all gases and ions (except for H_2_),^5^ is the thinnest (atomic layer) and most promising coating material for corrosion protection. It has been reported that the incorporation of a 30–40 nm graphene layer in polyethylenimine (PEI) as an additive in polymer-based corrosion protection coatings can dramatically reduce the oxygen permeability to 0.05 cm^3^/m^2^ per day.^6^ Recently, we have shown that layered composites of graphene oxide (GO) and PEI can be produced with nanometric thickness (<100 nm), achieving a 96% reduction in oxygen permeability, while pure PEI shows no barrier effect to oxygen.^7^ However, it is still controversial if graphene itself is efficient as a protective coating material to prevent the corrosion of metals. For example, it has been shown that a monolayer of graphene deposited on a Cu surface using chemical vapor deposition (CVD) does not protect the underlying Cu from oxidation; instead, it accelerates the corrosion of copper in the long term. This was attributed to the high conductivity of graphene, which can cause galvanic corrosion by forming an electrochemical circuit with the Cu, similar to contacting graphite with metal.^8^

The graphene/Cu interface plays a critical role in the oxidation and corrosion process. This is because the corrosion started at the graphene/electrolyte/Cu interface after the active gas and water penetrated through the defect. The horizontal diffusion of oxygen leads to severe corrosion of Cu.^9^ As an exception, the oxidation and corrosion of the underlying Cu can be completely inhibited by growing single-crystal graphene on Cu(111) as compared to Cu(100). This is because the strong interfacial coupling of the commensurate graphene/Cu(111) prevents H_2_O diffusion into the graphene/Cu(111) interface.^5^ However, it is challenging to achieve a commensurate large-scale graphene coating on arbitrary metals for practical applications. As an alternative, the multilayer strategy has been used, in which CVD graphene is stacked to form a relatively thick film to prevent the penetration of oxygen by forming tortuous channels. The multiple layers of CVD graphene can have a multiplicative effect and act as a better diffusion barrier than a single layer.^10^ However, it is rather complicated to transfer and stack CVD graphene without forming wrinkles and defects.^11^

Instead of using graphene to achieve a thin coating, silanes are a group of hybrid organic–inorganic compounds that can self-assemble into a single-layer film (nanometer thick) on any substrate.^12−14^ The silane monolayer coating is also known to tune the surface properties of substrates such as Si wafers, since the organic functional group on the other side of the silane can be hydrophobic or hydrophilic.^12,15^ Similar to the hydrolysis of silane on Si wafers, the silanol groups have the possibility to bond to the metal surface with strong adhesion.^16^ However, a monolayer silane coating with subnanometer thickness is not sufficient as an inhibition layer for the corrosion protection of metals because of the diffusion of aggressive ions and water. Therefore, instead of a monolayer, a thick and dense silane film is required as a barrier to protect the metal surface.^17^ Unfortunately, it has been shown that increasing the thickness does not guarantee the improvement of the corrosion protection because of the formation of pores in the thick silane film.^1^ The incorporation of silane into a polymer can not only dramatically improve the corrosion protection but also enhance the adhesion of the polymer–silane hybrid coating on the metal substrate.^18^ However, pore formation cannot be avoided when the solvent evaporates, which means that aggressive agents can penetrate through the micropores. Therefore, a new coating strategy would be needed to combine the benefit and achieve synergy among these different advanced coating materials (e.g., silane, polymers, and graphene).

**Scheme 1:**
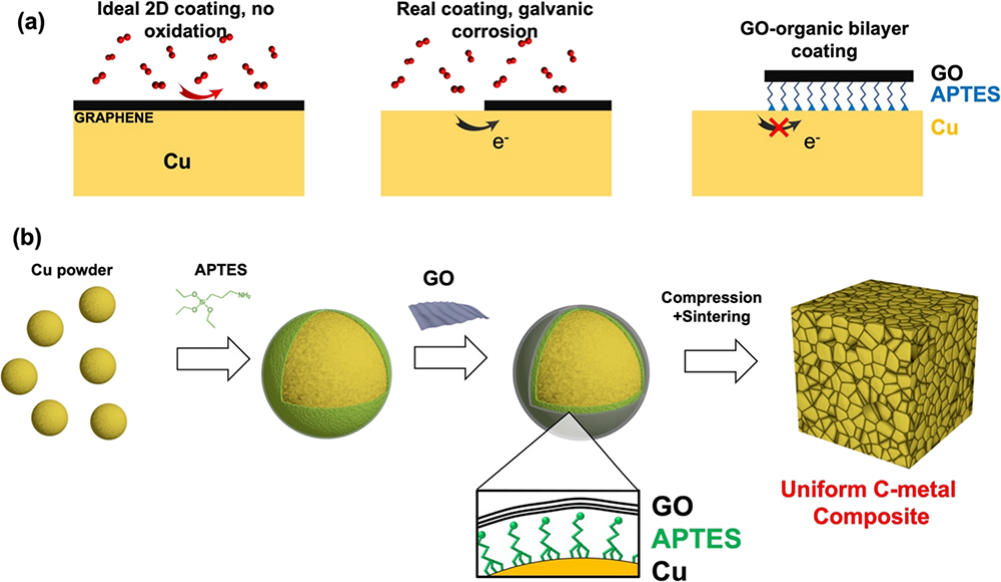
(a) Schematic Illustration of (Left) the Graphene Coating as a Protection Layer for the Gas Permeation, (Middle) Defect on the Graphene-Coated Cu, and (Right) APTES as an Insulating Layer to Prevent the Direct Contact of GO with Cu. (b) Schematic Illustration of the Synthesis Process of GO-Containing Cu

In this study, to avoid the direct contact of highly conductive reduced graphene oxide (RGO) with Cu, an insulating silane layer with a controllable thickness was inserted between GO and Cu (Scheme 1a).

This is a win–win strategy as a double protection coating, which complements the poor barrier properties of silane against aggressive agents by integrating the RGO barrier layer, resulting in a spatial separation of RGO and Cu (Scheme 1). Benefiting from the atomic layer of RGO and the formation of the monolayer of silane, the resulting RGO/silane protective coating can be very thin. The (3-aminopropyl)triethoxysilane (APTES) (−NH_2_ is the terminating group of the organic side) was selected and covalently bonded to the surface of Cu particles through the formation of Si–O–Cu, which led to strong adhesion. Its thickness increased with an increasing concentration of APTES due to the hydrolysis and self-polymerization of silanes. The negatively charged GO sheets rapidly self-assembled on the surface of APTES-coated Cu particles, forming a multilayered composite coating (GO-A-Cu) on the particles in the aqueous solution due to the electrostatic interaction. The GO coating could be tuned going from partial to full coverage, up to a relatively thick coating by adjusting the thickness of APTES or the loading of GO. The evolution of the surface coating was systematically monitored by scanning electron microscopy (SEM), Raman, X-ray photoelectron spectroscopy (XPS), and thermogravimetric analysis (TGA). After coating, the Cu powders were compacted and further sintered to obtain bulk samples and then tested for corrosion. Several studies showed methods to deposit Cu particles on the surface of GO;^19^ here, we use an analogous approach to do the opposite, i.e., to deposit GO on the surface of Cu microparticles.

Thanks to this dual coating, Cu coated with GO/APTES as well as with RGO/APTES showed improved corrosion protection in the concentrated acetic acid and ammonium persulfate. The potentiodynamic polarization measurements show that presintered GO-A-Cu features a lower corrosion current and more positive corrosion potential.

## Results and Discussion

### Controllable Coating Silane and GO on the Surface of Cu Particles

**Figure 1:**
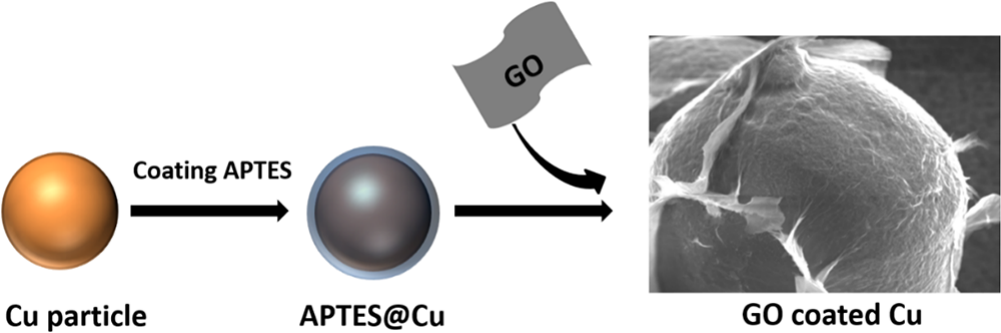
Synthesis procedure of GO-coated Cu particles.

The as-received commercial Cu particles from Carpenter Powder Products AB were used directly without further treatment for coating of APETS and GO (Figure 1). Since the Cu particles were stored in an ambient atmosphere after manufacture, a thin layer of CuO/Cu(OH)_2_ (eq 1) (as demonstrated by XPS; see below) formed on their surface. This layer can be further oxidized to Cu_2_O/CuO if the particles are stored in dry air (eq 2).^20,21^
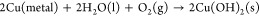
1
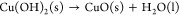
2

This oxidation process is of course much more relevant for Cu micropowders as compared to bulk Cu due to the much larger surface area. The average diameter of the Cu particles used in this study is about 50 μm but with broad size variation from 1 to 90 μm. The size of the Cu particles is much larger than the lateral size of GO sheets (from a few nm to a few μm). Hence, one of the aims of this study is to coat micrometer-sized Cu particles with GO.

The original Cu particles possess a spherical shape with a smooth surface and no signs of surface contamination. Due to the poor interaction, GO and Cu particles cannot be assembled together by simple mixing in solution (Figure S1).^22^ To increase the interaction, either the surface of Cu or GO needs to be modified. APTES was chosen as a linker because it is environmentally friendly and a commonly used silane to modify the surface of various materials, including metal oxides, metals, and polymers.^12,15^

**Figure 2:**
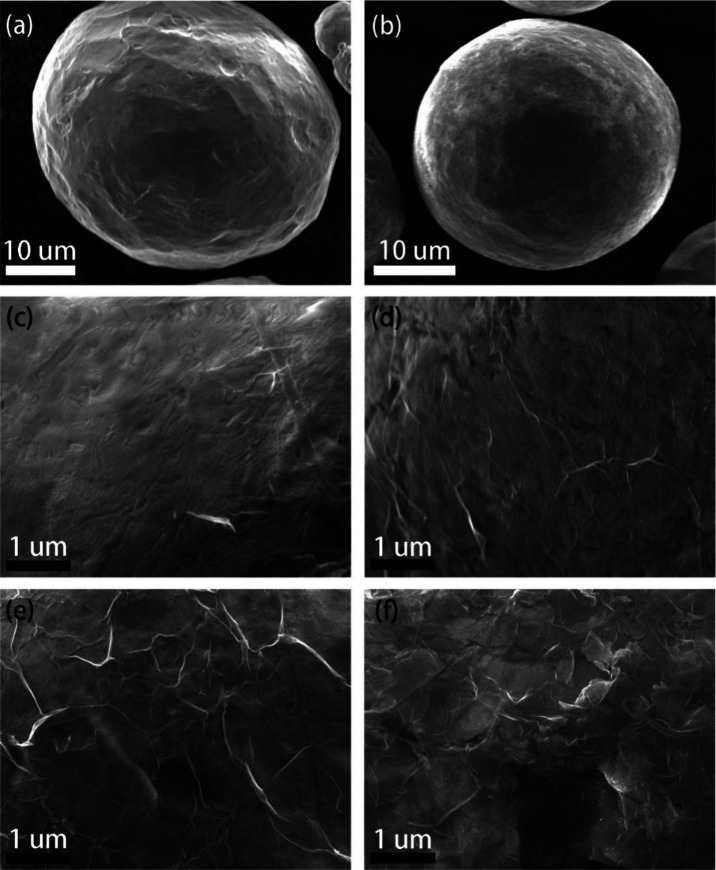
SEM images of (a) as-received Cu particles, (b) APTES-coated Cu particles, and GO-A-Cu synthesized using various concentrations of APTES solutions: (c) 0.05%, (d) 0.2%, (e) 0.8%, and (f) 1.5%.

A thin layer of APTES molecules was first covalently grafted onto the surface of Cu by forming the Si–O–Cu bonds, which ensures the strong adhesion of APTES on Cu particles (APTES-Cu).^12^ With different concentrations of APTES (0.05, 0.1, 0.2, 0.5, 1, and 2.5%), further condensation leads to the self-polymerization of APTES and to an increase of the silane thickness. After coating with APTES, the Cu particles show increased roughness as shown in the SEM images (Figure 2b and Figure S2).

The thickness of APTES increases with increasing APTES concentration, in accordance with a previous report.^23^ The amino functional group on APTES makes the Cu particles positively charged in acidic aqueous solution.^12^ The GO nanosheet is negatively charged due to the presence of carboxylic groups at the edge of the sheets (Figure S3).^24,25^ The strong electrostatic interaction allows the rapid assembly of GO on the surface of APTES-Cu in just a few seconds, forming GO-A-Cu, as can be seen even with bare eyes observing by the change of the GO dispersion color due to the adsorption of GO on Cu particles (Video S1). The commercial GO used as the starting material was also characterized by Raman, XRD, and TGA (Figure S4).

**Figure 3:**
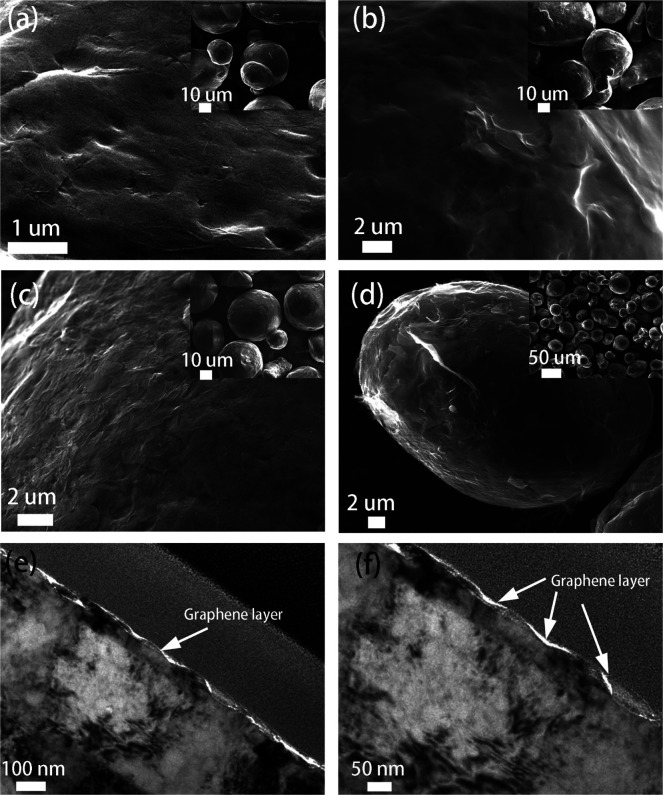
SEM and high-resolution TEM images of GO-A-Cu particles synthesized with different GO loadings: (a) 0.02 wt %, (b) 0.04 wt %, (c) 0.0625 wt %, and (d) 0.25 wt %. Inset: the corresponding low-magnification SEM images. (e, f) High-resolution cross-sectional TEM images of the GO-A-Cu with a GO loading of 0.0625 wt %.

The thickness of coated GO can be controlled by varying either the thickness of APTES or the loading of GO, as shown in Figure 1 (step two). The effect of these two experimental parameters on the thickness of the GO coating was systematically investigated. At an extremely low concentration of APTES (i.e., 0.05%), the APTES coating is very thin and cannot cover the entire surface of the Cu particles (Figure 2c). This leads to a partial coating of GO due to the low density of −NH_2_ groups on Cu, which cannot absorb enough GO to completely wrap Cu, even if in the presence of a high GO concentration. It is possible to discriminate the GO coating from the bare APTES-functionalized Cu by observing the pattern of ripples and sheet edges (Figure 2), typical of 2D materials deposited on a surface. When the concentration of the APTES solution was increased to 0.2%, the Cu was completely covered with an APTES film and further wrapped by a thin layer of GO (Figure 2d). Note that the GO coating is very thin, which even allows the underlying APTES film to be observed in the SEM. With further increase of APTES concentration to 0.8%, a thick GO film with numerous wrinkles can be observed on the surface of the APTES-Cu particle (Figure 2e). Thanks to the tendency of GO sheets to form robust multilayer stacks, the GO film adhered firmly to the surface of APTES-Cu after drying.^12,15^ When the APTES concentration reached 1.5%, a very thick APTES film was formed on the Cu surface. In summary, to achieve a uniform GO coating and a fairly strong adhesion, the optimized APTES concentration is between 0.2 and 0.8%. Note that this approach achieved a dense coating not only on the large Cu particles but also on small particles of debris with irregular shapes (inset of Figure 3). This implies that the coating process is not limited to the specific size and geometry of particles, demonstrating that our method is a universal process for the controllable coating of metal particles with GO.

The effect of GO loading on the coating thickness was further investigated. The synthesized APTES-Cu particles were washed thoroughly with toluene and water to remove unreacted molecules and ensure the formation of an even, thin APTES layer on the Cu surface. Then, they were mixed with dispersions containing GO at different concentrations, which was quickly adsorbed on the Cu surface as mentioned before. After adding different amounts of GO, the evaluation of the surface morphologies of GO-A-Cu was investigated by use of SEM (Figure 3). In the case of an extremely low GO loading (0.02 wt %, as calculated on the total mass of the sample) (GO(0.02 wt %)-A-Cu), all the added GO nanosheets were absorbed on the surface, but they were not enough to cover the entire surface of Cu particles, which led to a partial coating (Figure 3a). Since the Cu surface was completely covered with APTES, as demonstrated in the previous session (Figure 2), part of the Cu surface was coated with APTES but no GO can be observed in the SEM (Figure 3a). With a higher GO loading of 0.04 wt % (GO(0.04 wt %)-A-Cu), the Cu particles were completely covered by GO sheets (Figure 3b), yielding a smooth surface with the presence of GO wrinkles. The coatings are sufficiently thin to discern the features of the APTES-Cu particle, indicating that the adsorbed GO is only a few layers thick. When the GO loading reaches 0.0625 wt % (GO(0.0625 wt %)-A-Cu), the presence of numerous wrinkles indicates that the stacked multilayer GO completely wrapped the ATPES-Cu particle (Figure 3c). The coating is very uniform. All of the Cu particles were isolated, and no GO clusters were observed, as shown in the inset SEM image of Figure 3c. At a higher GO loading (0.25 wt %) (GO(0.25 wt %)-A-Cu), saturation of the surface was reached, and not all the GO present in solution was adsorbed. The particles showed a thick layer with clearly visible edges and wrinkles (Figure 3d); some of the coated GO sheets are partially peeled off from the surface of APTES-Cu.

The stacked layer structure of GO on the surface of Cu was further investigated by high-resolution TEM (Figure 3e,f). A TEM sample from a single Cu particle coated with GO was produced by the focused ion beam lift-out technique. The cross-sectional TEM images confirmed the complete coating of GO along the surface of the Cu particles. The coated GO layers are very thin. Unfortunately, we are not able to measure the thickness of coated GO based on the TEM images because the flexible GO layers are too thin and folded.

**Figure 4:**
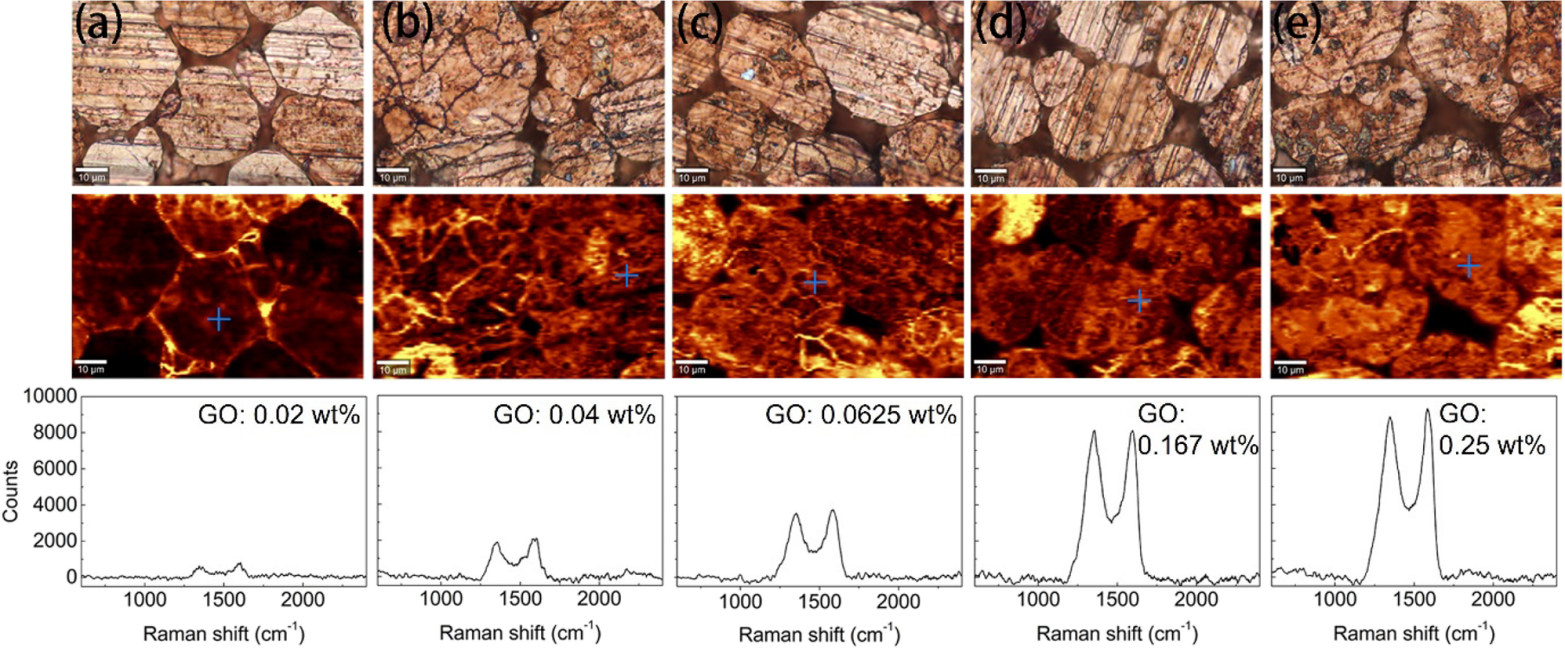
Optical microscopic images (first row), Raman contrast mapping (second row), and single Raman spectra (third row) of compressed GO-A-Cu powders synthesized under different GO loadings: (a) 0.02 wt %, (b) 0.04 wt %, (c) 0.0625 wt %, (d) 0.167 wt %, and (e) 0.25 wt %.

The compressed GO-A-Cu samples were further studied by using Raman spectroscopy (Figure 4). Two Raman characteristic peaks of GO (e.g., D band and G band) at ∼1360 and ∼1580 cm^–1^ can be observed in all GO-A-Cu samples, confirming the presence of the GO coating.^26,27^ The Raman maps are displayed with respect to the intensity of the G band at ∼1580 cm^–1^ (Figure 4, middle images). In addition, a single spectrum was recorded for each sample at the point of interest in the Raman maps (Figure 4, bottom row). The particles can be readily recognized in the Raman maps, thanks to the uniform coverage of GO. The boundaries between adjacent particles are brighter due to the significant thickness of the GO coating. A darker area can be seen in the Raman map of GO(0.02 wt %)-A-Cu, which indicates that there, the surface is not covered by GO. This is in agreement with the SEM results, which showed only partial coverage for GO(0.02 wt %)-A-Cu (Figure 3b). The increased intensity of the G band (Figure 4, bottom row) indicates the increased thickness of the coated GO, since the intensity of the G band is proportional to the number of GO layers.^28,29^ The GO(0.25 wt %)-A-Cu and GO(0.167 wt %)-A-Cu show similar intensities in the Raman maps. This is because the GO coating is saturated.

**Figure 5:**
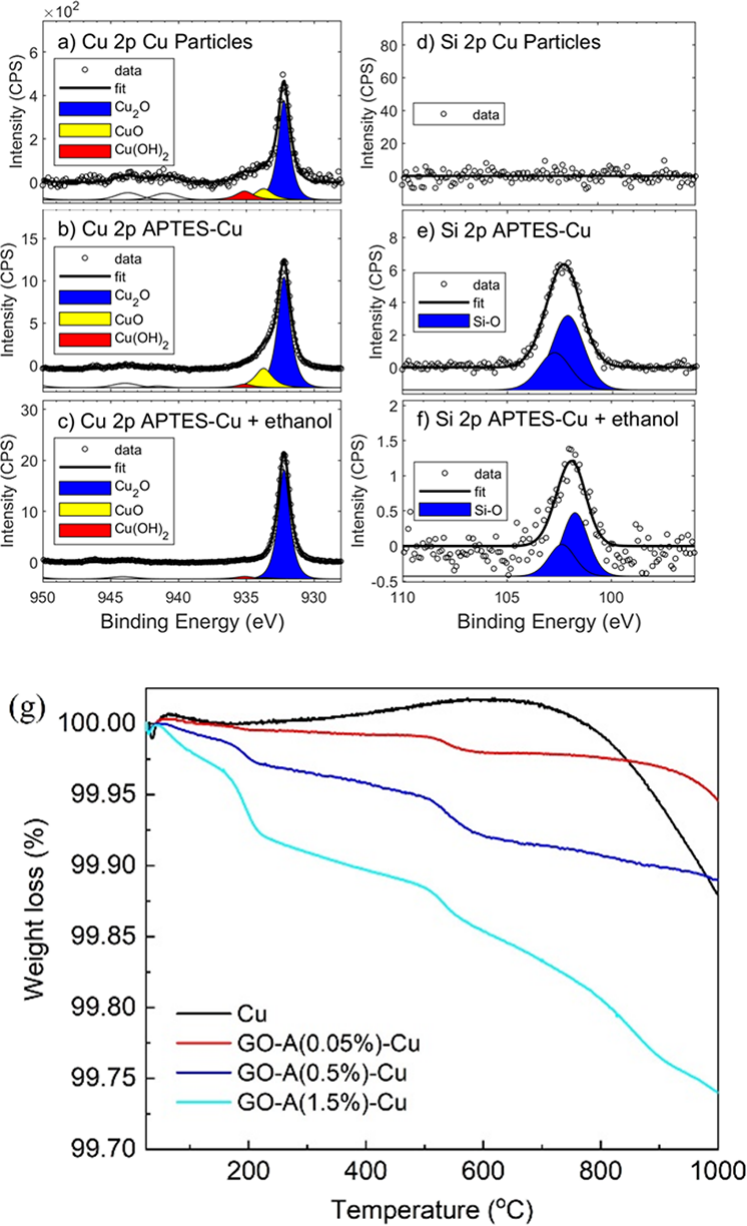
Cu 2p XPS spectra of (a) pure Cu particles, (b) APTES-Cu synthesized using 1% APTES solution, and (c) APTES-Cu after rinsing in ethanol. Si 2p XPS spectra of (d) pure Cu particles, (e) APTES-Cu synthesized using 1% APTES solution, and (f) ethanol-washed APTES-Cu. (g) TGA curves of Cu, GO-A(0.05%)-Cu, GO-A(0.5%)-Cu, and GO-A(1.5%)-Cu.

The strong adhesion of APTES and GO can be attributed to the covalent bonding of APTES to the Cu surface and the strong electrostatic attraction between APTES and GO at corresponding two interfaces. The formation of Si–O–Cu bonds was indirectly confirmed using XPS spectroscopy (Figure 5 and Figures S5 and S10). As aforementioned, the surface of Cu particles consists in a CuO oxide with a thin layer of Cu(OH)_2_ and Cu_2_O coexist on the surface of the Cu particles, which was confirmed by the presence of Cu 2p_3/2_ peaks at 935.2 eV (Cu(OH)_2_) and 933.7 eV (CuO) in the Cu 2p XPS spectra of the initial Cu particles (Figure 5a).^21^ The main phase of CuO was confirmed by the Auger modified parameter (1849.2 ± 0.1 eV in all samples; see Figure S10) and the Cu 2p_3/2_ peak at 932.2 eV.^30,31^ A condensation reaction occurs between the −OH at the surface of Cu and the OH groups of the hydrolyzed APTES.^1,16,18^ The peak corresponding to Cu(OH)_2_ drastically decreases from 4.9 to 0.5% after the coating with APTES, which can be ascribed to the formation of Cu–O–Si (Figure 5b). This peak was retained even after the APTES-Cu particles were thoroughly rinsed with ethanol (Figure 5c). The decrease in intensity after washing indicates the presence of weak APTES molecules adhering via hydrogen bonds in addition to the covalently bonded APTES. Consistent with the evolution of the Cu–O–Si bond, the Si 2p peak appeared after APTES was applied and remained after washing with ethanol (Si 2p_3/2_ at 102.1 eV in Figure 5d–f). This finding shows the presence of covalent bonds at the interface between Cu and APTES, which ensured the strong adhesion of the coating. The presence of the Cu peak after the coating of APTES and GO confirms that the thickness of GO coating is very thin, considering that the depth of the XPS measurement is about 5–10 nm.

The GO surface is negatively charged with a zeta potential of −34 mV due to the presence of various C–O groups (e.g., carboxylic group).^15,32,33^ After treatment with APTES, the zeta potential of GO increased to 3 mV, indicating the strong electrostatic interaction between GO and APTES (Figure S3). Accordingly, APTES-coated Cu particles feature −NH_2_ groups with a positively charged surface. Thanks to the strong electrostatic attraction, the GO nanosheets can thus self-assemble on the APTES-Cu surface in a matter of seconds (Video S1). After drying, the coated APTES is further cross-linked through a condensation reaction, removing water molecules. Also, the water molecules intercalated between GO layers were partially removed, yielding a dense and strongly adhering GO/APTES coating on Cu (Figures 1–3).^32^

Further information about the chemical composition and thermal stability of GO-A-Cu can be obtained from TGA (Figure 5g).^34^ The original Cu shows a slight mass loss of about 0.1 wt % from 700 until 1000 °C connected to the partial dissociation of copper oxide. All GO-A-Cu samples show two steps of weight loss at ∼195 and ∼550 °C, corresponding to the removal of oxygen-containing functional groups from GO and the further condensation/decomposition of APTES, respectively.^35^ The APTES-related second step in weight loss increased from 0.01% for GO-A(0.05%)-Cu to 0.03% for GO-A(0.5%)-Cu and further to 0.04% for GO-A(1.5%)-Cu. As the concentration of APTES increased, the slightly increased weight loss derived from APTES at ∼550 °C implied an increased coating thickness of APTES.

With increasing APTES concentration during synthesis, the weight loss of the GO component (∼200 °C) due to the reduction of GO in the GO-A-Cu samples increased proportionally. In agreement with the SEM observations (Figure 2c), for the GO-A(0.05%)-Cu, a negligible weight loss (0.003 wt %) can be observed at ∼200 °C due to the partial coating of GO (note: the loading of GO is very low). As the concentration of APTES increased to 0.5%, the weight loss of the GO component also increases to 0.02 wt %, confirming the increase of the GO thickness. The weight loss reaches 0.05 wt % when the 1.5% APTES concentration was used for the synthesis.

Based on the thermal behavior observed in TGA, heat treatment of GO-A-Cu at 400 °C was performed. At this temperature, GO is reduced to more conductive RGO (RGO-A-Cu), while APTES molecules condense by releasing water;^36^ the heat treatment also removes the inserted solvent molecules between the GO layers, resulting in a reduced interlayer distance, as evidenced by the shift of the (002) diffraction peak from 11.8 and 23.5° (Figure S6).^7,24,32^ This could be beneficial for blocking the diffusion of oxygen and ions from the electrolyte/RGO interface to the Cu/APTES interface via the interlayers. XPS also confirmed the reduction of the GO component in the GO-A-Cu after the thermal treatment at 400 °C by a significant decrease of oxygen (Figure S12). After thermal treatment, a uniform RGO film can be clearly observed on the surface of the Cu particles. The Cu particles are fully covered with RGO without exposing the underlying surface, which could provide better anticorrosion protection. Raman contrast mapping of the RGO G band confirms that the RGO-A-Cu powders remain fully coated with RGO after reduction (Figure S7).

Further on, the RGO-A-Cu samples were compacted and sintered. Although the Cu particles deform during compaction, no cracks or fractures are observed on the RGO film, thanks to the high mechanical properties and flexibility of RGO.

Composites by simple mixing of GO and Cu were also prepared to be used as reference samples. Also, in these composites, the amount of GO was varied. In these conventional composites, the GO tends to agglomerate (see Figure S1).

**Figure 6:**
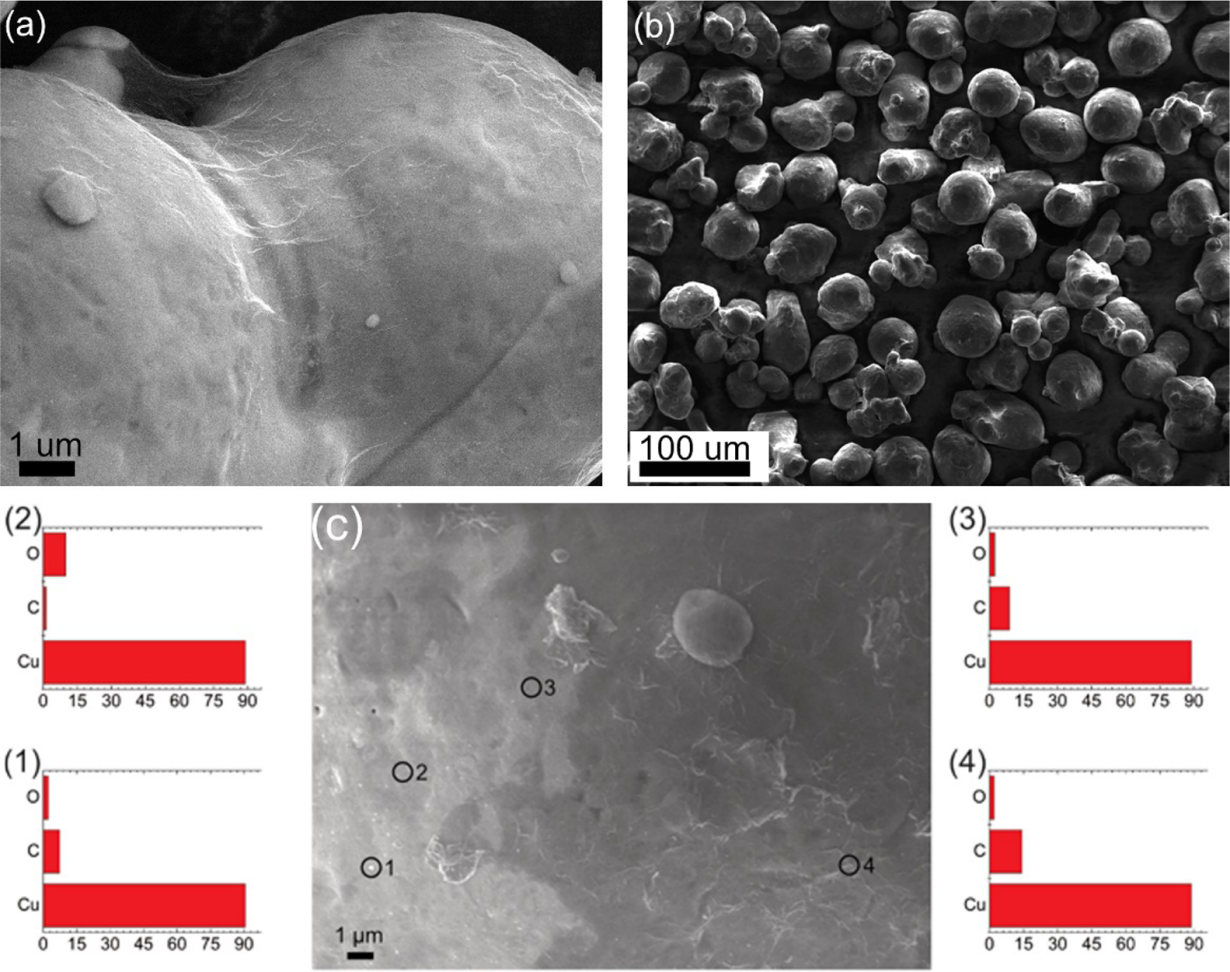
SEM images of the RGO(0.0625%)-A-Cu particle at (a) high and (b) low magnifications. (c) High-resolution SEM image of an RGO-A-Cu particle synthesized using 0.02 wt % APTES solution and EDX analysis (points 1–4: chemical composition obtained by EDX analysis), showing that the APTES-Cu was partially coated with RGO.

Figure 6c shows an HR-SEM image of the surface of an RGO(0.04%)-A-Cu particle. This particle was partly coated with GO prior to the reduction. The area coated with RGO and the uncoated area can be easily distinguished. Such a partially coated area allows us to compare the composition of both the APTES-coated Cu and the RGO-coating Cu by EDS. The inset histograms 1 to 4 show the elemental composition at the corresponding points in the SEM image. Points 1 and 2 show mostly Cu and small amounts of C and O, which was derived from the APTES. At points 3 and 4, the C-content increased, confirming the presence of RGO. The low oxygen content indicates that the GO component was well reduced.

After reduction, the RGO-A-Cu powder was grinded. Although the Cu particles were strongly deformed, the RGO could still be observed attached (Figure S14), indicating its strong adhesion to the surface of the Cu particles.

**Figure 7:**
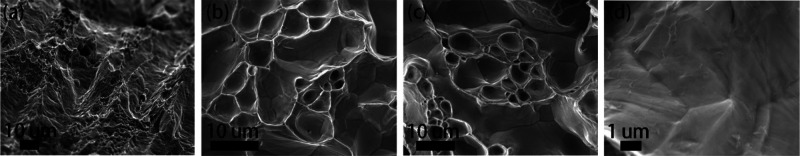
SEM images of the fracture surfaces of sintered (a) Cu, (b) RGO(0.02 wt %)-A-Cu, (c) RGO(0.04 wt %)-A-Cu, and (d) RGO(0.25 wt %)-A-Cu.

The compacted RGO-A-Cu was further annealed at 1030 °C to presinter the particles. Figure 7a shows a typical fracture surface of pure Cu, with a clearly pronounced interparticle ductile fracture with fine dimples. A similar structure but with smaller domains of the ductile fracture was found in partially and thinly RGO-coated samples (Figure 7b,c), where the boundaries of adjacent Cu particles can be observed in these samples. The fracture surfaces of the coated samples show the lower fracture of the ductile fracture with the presence of the extended areas of unsintered powder surfaces, which could be beneficial for certain applications where a protection layer is needed.^37^ Although the boundaries of particles present on the fracture surface, the RGO are hardly observable in the SEM image due to the thin coating. The EDX elemental mapping shows a high content of C and some Si, confirming the presence of RGO and decomposed APTES after high-temperature annealing (Figures S8 and S9). For samples with a relatively thick RGO coating (e.g., RGO(0.4%)-A-Cu) (Figure 7d), the RGO layer can be found on the fracture surface of Cu. The thick layer of APTES and RGO hampers the sintering of Cu particles, resulting in partially isolated particles even after high-temperature sintering.

**Figure 8:**
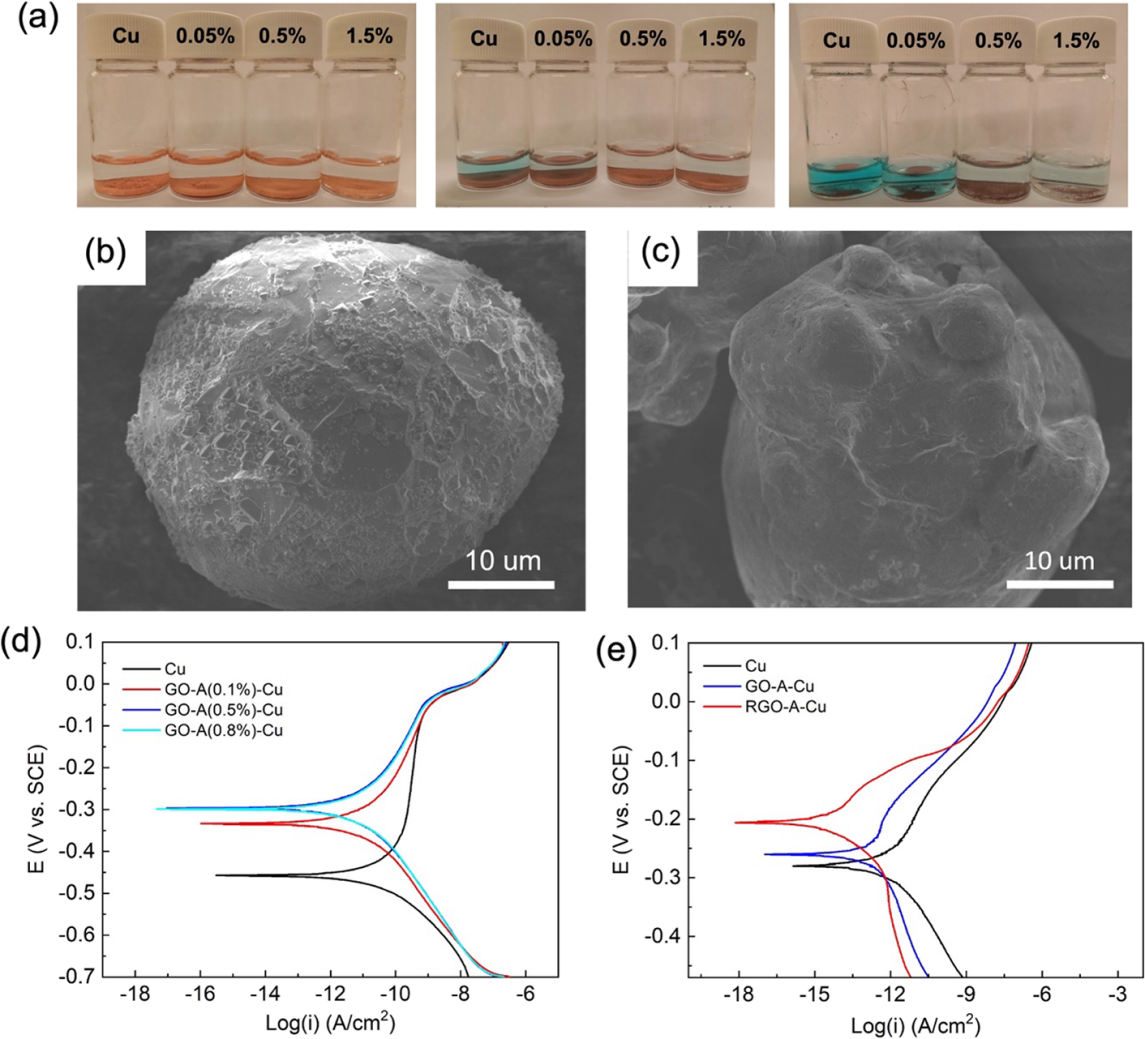
(a) Optical images of (1) Cu, (2) RGO-A(0.05%)-Cu, (3) RGO-A(0.5%)-Cu, and (4) RGO-A(1.5%)-Cu in 10% acetic acid after (from left to right) 0, 19, and 99 h. SEM images of (b) pure Cu particles and (c) RGO-A-Cu particles after soaking in the acetic acid after soaking for 19 h. (d) Tafel curves of compressed Cu and GO-A-Cu synthesized with various concentrations of APTES. (e) Tafel curves of Cu and sintered GO-A-Cu and RGO-A-Cu composites.

To evaluate the corrosion protection provided by silane and the RGO, Cu and RGO-A-Cu (concentrations of APTES: 0.05, 0.5, and 1.5%) were immersed in 10 mL of acetic acid (10% in water) for 99 h. It is known that Cu does not react with acetic acid. However, in the presence of oxygen, the reaction accelerated with the formation of green Cu(II) acetate. Such an experiment examines not only the protective effect of APTES against the acid but also the permeability properties of the stacked RGO layers to oxygen gas. As shown in Figure 8a, the acetic acid solution with Cu turned blue after 19 h, indicating the corrosion of Cu/Cu oxides by the acetic acid. A lighter blue color could be observed in the acetic solution containing RGO-A(0.05%)-Cu. The partially coated APTES and RGO cannot provide complete protection of the Cu surface, resulting in corrosion in the area where the Cu surface is exposed. In comparison, the color of the acetic solutions with RGO-A(0.5%)-Cu and RGO-A(1.5%)-Cu did not change significantly. This is mainly because the permeation and diffusion of oxygen and acetic acid were blocked by a stacked RGO and APTES coating. Even after 99 h, RGO-A(0.5%)-Cu remained protected. However, the color of the acetic solution containing RGO-A(1.5%)-Cu began to turn to blue. The poor performance of the 1.5% sample could be attributed to the excessive thickness of the GO, causing poor adhesion and delamination among the particles and creating preferential paths for penetration of the acid. The Cu particles with only APTES coating also showed improved anticorrosion performance as compared with pure Cu, in agreement with the previous report.^23,38^ The color of the acetic acid solution changed slightly (Figure S11). However, due to the presence of pores and the slow diffusion of aggressive etchants, the pure silane coating was not good enough for long-term anticorrosion protection. Also, the RGO-A-Cu in degassed acetic solution did not change after 24 h of soaking (Figure S13), indicating the good barrier property of multilayer RGO coating on the surface of Cu.

The morphologies of the pure Cu particles and RGO-A-Cu particles after soaking in 10% acetic acid were also investigated (Figure 8b,c). The pure Cu particles showed a heavily corroded surface, featuring pyramidal etching features due to preferential etching (Figure 8b). Conversely, no such features were observed on the RGO-A-Cu particles after soaking, which implies that the Cu surface was not affected by acetic acid due to the surface protection and barrier role of RGO (Figure 8c).

The galvanostatic polarization was used to evaluate the anticorrosion performance of RGO-coated Cu, exposed to a solution of 3.5 wt % NaCl. Prior to measuring the samples, the potentiodynamic cells were equilibrated for 20 min to reach stable open circuit potential. Figure 8d shows the Tafel curves for compressed Cu and GO-A-Cu composites with various APTES loadings, considering that the APTES also plays a role in the anticorrosion. Compared to compacted Cu powder, all GO-A-Cu green bodies exhibit a corrosion potential *E*_corr_ more positive than the initial −0.46 V and reduced cathodic polarization potentials. These results indicate that the kinetics of the anodic reactions Cu(s) → Cu^2+^+ 2e^–^ and Cu(s) → Cu^+^+ e^–^ have not been significantly prevented by the coating of APTES and GO. On the other hand, reduction reactions on the Cu surface were inhibited. This shift in corrosion potential correlates with the thickness of APTES and GO coating as indicated by a +0.12 V shift for 0.1% APTES and a +0.15 V for 0.8% APTES. The GO-A-Cu composite powders synthesized with 0.5 and 0.8% APTES exhibit a slightly higher positive shift, which suggests that the cathodic corrosion inhibition is not dominated by APTES coverage alone.

These results show that the dual protection of APTES and GO on Cu powder inhibits the cathodic corrosion reaction since it acts as an oxygen diffusion barrier. The corrosion protection behavior for sintered GO-A-Cu composites was also investigated and compared with that of sintered Cu. The Tafel curves are shown in Figure 8e. It can be seen that reduction of GO-A-Cu prior to sintering is important for retaining a protective layer on the composite surface. The improved anticorrosion performance of sintered GO-A-Cu as compared to Cu indicates the presence of RGO on the surface of Cu after high-temperature sintering, even if the RGO coating is thin. The anticorrosion performance of sintered RGO-A-Cu dramatically improved due to the sufficient passivation to result in a +0.9 V shift in *E*_corr_ to −0.206 V and a reduction of the corrosion current *i*_corr_ from 7.14 × 10^–6^ to 3.86 × 10^–7^ A/cm^2^.

Thermal reduction of the coated particles has a significant effect on the polarization kinetics. The anodic domain in RGO-A-Cu (i.e., the upper branch of the red curve in Figure 8e) displays a passivated regime that is absent in the samples of Cu and GO-A-Cu (black and blue lines in Figure 8e, respectively), resulting in log(*i*) only changing by 0.5 between −0.45 V < *E* < −0.27 V. Hence, within the interval −0.45 to −0.3 V, the log(*i*) for the Cu sample changes nearly four times. In addition, log(*i*) in the cathodic domain between −0.206 and −0.07 V is initially much lower than for both the Cu and GO-A-Cu samples. However, as shown in Figure 8e, the asymmetric polarization curve for RGO-A-Cu indicates that there is passivation for the cathodic reaction. The RGO coating can passivate the sample surface to limit the anodic decomposition of Cu and the cathodic reaction involving reduction of dissolved oxygen. However, the cathodic reduction of oxygen is diffusion limited. The polarization curves of pure Cu and RGO-A-Cu were further fitted to estimate the corrosion current density *i*_corr._ The results show that the corrosion current density *i*_corr_ of RGO-A-Cu (1.91 × 10^–5^ A/cm^2^) is lower than that of Cu (7.14 × 10^–5^ A/cm^2^), indicating improved anticorrosion performance.

## Conclusions

While there are several works demonstrating the beneficial use of nanomaterials for anticorrosion coating, our approach targets successfully the issue of galvanic corrosion often caused by such coatings; furthermore, it demonstrates that such a nanocoating approach is effective not only on the metal particles but also in the final material that shall be obtained by processing the particles, which is a major advance to use such coatings in real technological applications. The dual coating of silane and RGO works truly as a nanocomposite, where each component has a different role.

The stacked RGO acts as a gas barrier and blocks the diffusion of both oxygen and ions. The silane layer with a nanometer thickness serves both as a linker to bind GO on the surface of micrometer-sized Cu particles and as an insulating interlayer to prevent the electrochemical corrosion of Cu particles. The GO can either partially or completely coat the Cu particles. The thickness of GO was controlled by tuning the thickness of the silane interlayer and/or the loading of GO. The RGO was retained after high-temperature sintering. The anticorrosion tests demonstrate that the dual coating could effectively prevent corrosion of Cu under an aggressive acid and saline environment. The synthesis of the GO coating presented in this study is easily scalable and allows the thicknesses of both the silane layer and the GO coating to be adjusted. In addition, this work develops an efficient strategy to eliminate the galvanic corrosion effect of graphene, which opens a new avenue to improve the anticorrosion of metal products by taking advantage of the barrier properties of graphene.

## Experimental Section

### Synthesis of APTES-Cu Particles

The GO used as starting materials was received from Graphenea, Abalonyx, and LayerOne. The commercial Cu particles were used as the starting materials for the coating. In a typical coating procedure, 100 g of Cu particles was mixed with 100 mL of toluene. A certain amount of APTES was then added to the solution to achieve various concentrations: 0.05, 0.2, 0.5, 0.8, and 1.5%. The mixture was then vigorously stirred at room temperature for 16 h. After the supernatant solution was poured out, the APTES-coated Cu particles were washed with fresh toluene solution and water. The APTES-Cu particles were obtained after drying under vacuum.

### Synthesis of GO-A-Cu Particles

The synthesis procedure of the GO-A-Cu comprises two steps, as illustrated in Figure 1. The commercial GO dispersion (0.4 wt %) was diluted. After sonication, it was redispersed before use for the coating. The sonication process is important to exfoliate the aggregated GO. The well-dispersed GO dispersion with different loadings (0.02, 0.04, 0.0625, and 0.25 wt %) was then added to the APTES-Cu suspension under stirring. GO-A-Cu was then obtained after drying at room temperature.

### Reduction of GO-A-Cu Particles

The GO-A-Cu samples with different GO thicknesses were thermally treated at 400 °C for 3 h in a tube furnace under an Ar atmosphere. The heating rate was 5 °C/min. The slow heating could avoid the explosive exfoliation of GO during thermal reduction.

### Sintering of RGO-A-Cu Particles

GO-A-Cu powders were first compressed into a 1.2 cm^–1^ diameter disk in a stainless steel mold under 1.25 GPa by a hydraulic press. The compressed samples were sintered at 1030–1050 °C for 2 h in an alumina tube furnace under Ar. The sintered RGO-A-Cu disk was directly used for the potentiodynamic polarization test.

### Corrosion Test and Electrochemical Measurements

To investigate the dual protection of RGO and APTES on the as-synthesized RGO-A-Cu, 1 g of Cu, RGO-A(0.05%)-Cu, RGO-A(0.5%)-Cu, and RGO-A(1.5%)-Cu were added to 10% acetic acid, respectively. The vials containing the above solutions were gently shaken at least three times a day. The photographs of the solution were taken after 19 and 99 h.

The corrosion resistance of the sintered samples immersed in 3.5 wt % NaCl solution was investigated using an electrochemical workstation. A conventional three-electrode electrochemical system was utilized, for which sintered samples, Pt wire, and saturated calomel electrode (SCE) were used as the working electrode, counter electrode, and reference electrode, respectively. After 30 min of stabilization, the polarization curves were measured at a sweep rate of 0.5 mV/s from −0.5 to 0.1 V vs SCE. The Tafel curves were obtained accordingly from the polarization curves.

### Characterizations

The XPS spectra were collected using a PHI VersaProbe III with a monochromatic Al *K*_α_ source (1486.6 eV); the spot size was 100 μm. The Multipak Spectrum was used for the analysis of the spectra. The binding energies were calibrated using the C 1s peak at 285 eV. A JEOL JSM-7800F Prime scanning electron microscope equipped with an EDS was used for imaging and chemical analysis. TGA was performed using a Mettler Toledo TGA/DSC3+ system at a heating rate of 5 °C min^–1^ under Ar using about 200 mg of Cu-based samples. The Raman spectra were recorded using a WITec alpha300 R confocal Raman spectroscope with a laser wavelength of 532 nm. TEM images were recorded with a FEI Tecnai T20 transmission electron microscope equipped with a LaB_6_ cathode and using an acceleration voltage of 200 kV; the TEM sample for the cross-sectional measurements was prepared by a focused ion beam technique using a FEI Versa3D LoVac DualBeam equipment.

## Supporting Information Available

Coating GO on Cu particles in solution (MP4)
SEM image of the GO/Cu mixture, APTES@Cu; zeta potential, XRD, TGA, and Raman of GO; XPS Si 2p spectra of sintered GO-A-Cu; XRD of GO before and after reduction; Raman mapping of RGO-Cu; SEM and EDS of sintered RGO-A-Cu; XPS and Auger spectra of GO-A-Cu; table of the XPS result of different GO- and RGO-coated Cu; image of silane-coated Cu before and after soaking in acid (PDF)
