# Supplementary material for: Controllable Coating Graphene Oxide and Silanes on Cu Particles as Dual Protection for Anticorrosion
Source: ACS Appl Mater Interfaces. 2023 Aug 7;15(32):38857–66. doi: 10.1021/acsami.3c08042 (PMC10436246; doi:10.1021/acsami.3c08042)
Supplement: Supplementary file 2 — am3c08042_si_002.pdf [file am3c08042_si_002.pdf]

Supporting Information

Controllable coating graphene oxide and silanes on Cu particles as dual protection for anticorrosion

Jinhua Sun[a,*], Kristoffer Harr[a], Uta Klement[a], Alessandro Kovtun[b], Zhenyuan Xia[a], Plinio Fernandes Borges Silva[a], Eduard Hryha[a], Lars Nyborg[a], Vincenzo Palermo[a,b,*]

[a]Department of Industrial and Materials Science, Chalmers University of Technology, SE-41296 Gothenburg, Sweden.

[b]Institute of Organic Synthesis and Photoreactivity (ISOF), CNR, via Gobetti 101, 40129, Bologna, Italy.

Corresponding author: jinhua@chalmers.se and palermo@chalmers.se

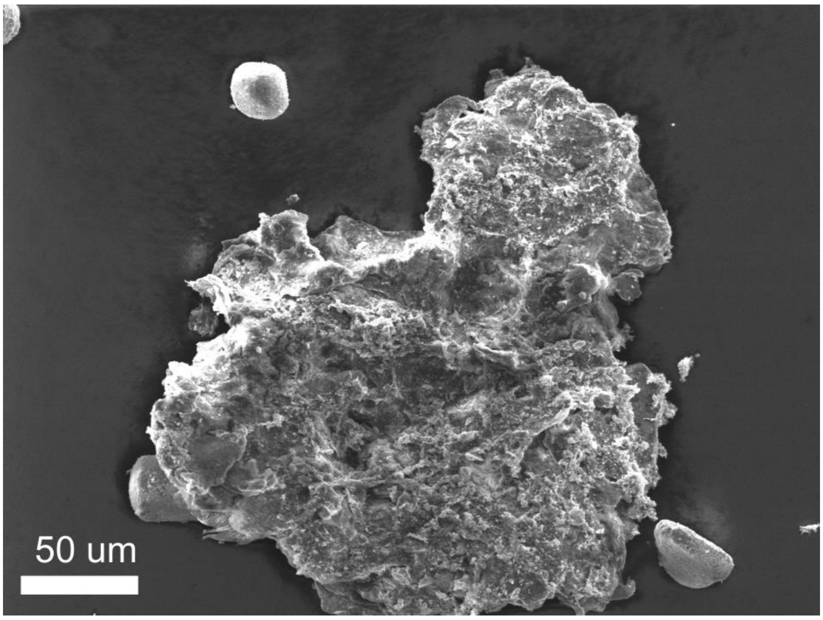

Figure S1. The SEM image of GO/Cu composite prepared by simply mixing Cu particles with GO solution. Aggregation of graphene can be observed, and the Cu was not coated by graphene.

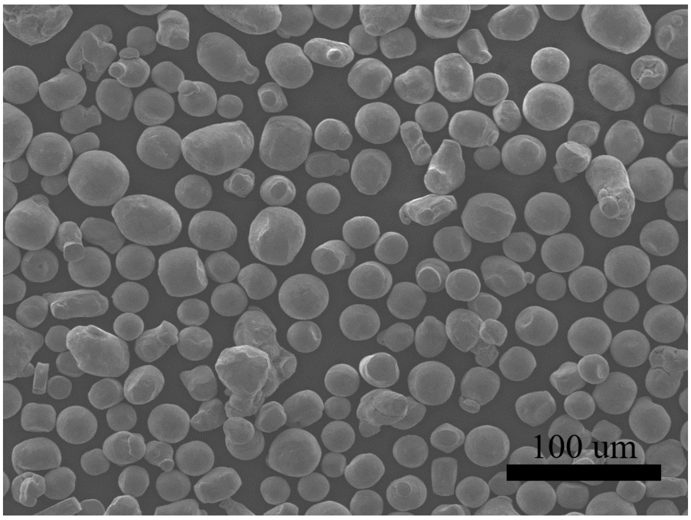

Figure S2. SEM images of APTES@Cu synthesized by 1.5% APTES.

Supporting information

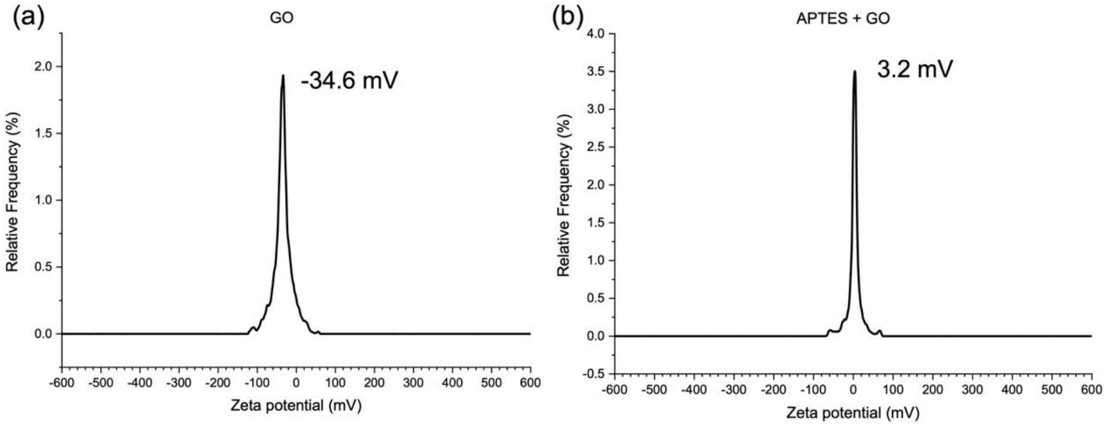

Figure S3. Zeta potential distribution of (a) GO dispersion and (b) the mixture of GO and APTES.

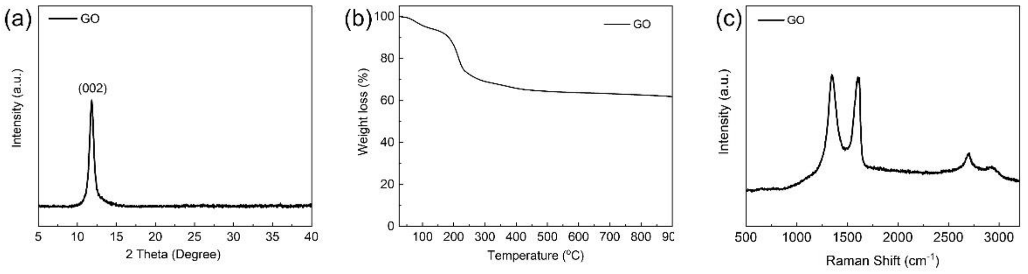

Figure S4. The (a) XRD pattern, (b) TGA curve, and (c) Raman spectrum of GO.

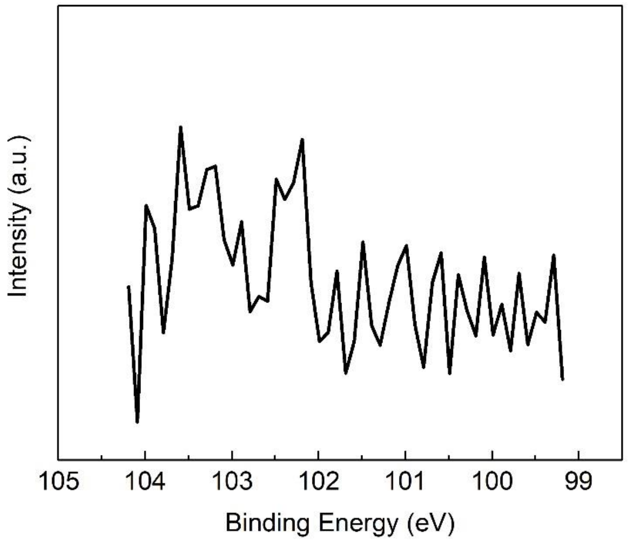

Figure S5. XPS Si 2p spectra of sintered GO-A-Cu synthesized using 0.5% concentration of APTES.

XPS is a surface technique analysis. Its detection depth is only few nanometers. The weak intensity of Si 2p peak confirms the good, uniform coverage of graphene, with the APTES molecules buried beneath the GO layers.

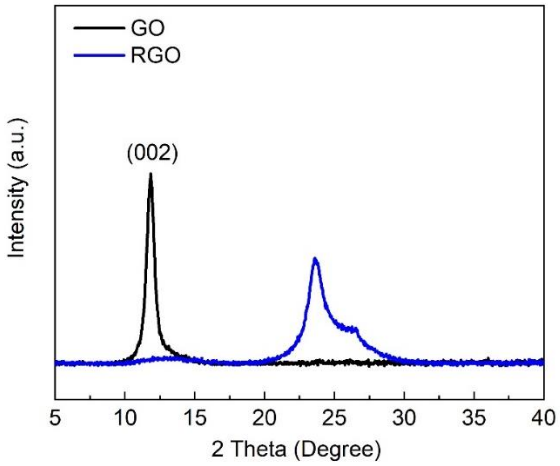

Figure S6. The XRD pattern of GO before and after thermal reduction.

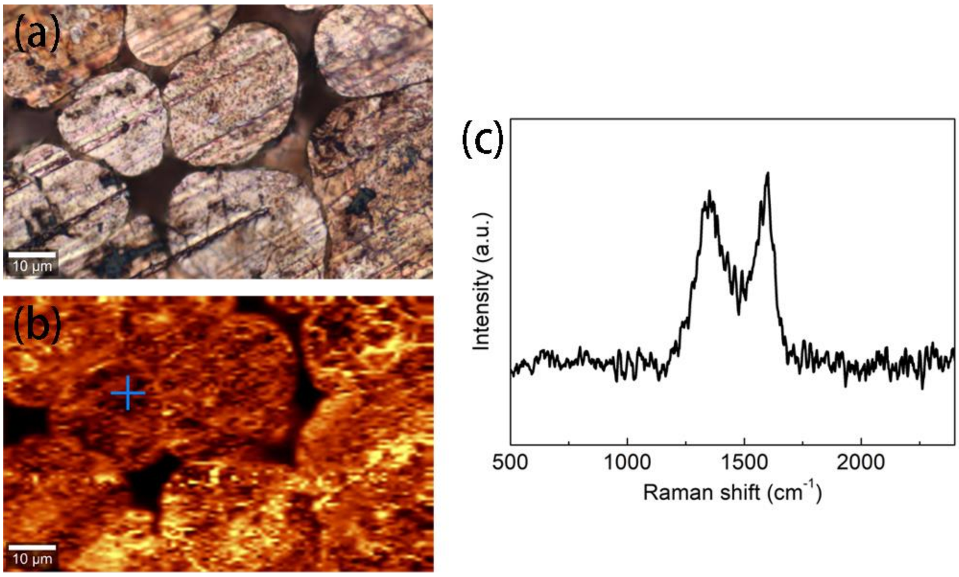

Figure S7. (a) Optical microscopic images, (b) Raman contrast mapping, and (c) single spectra of RGO-Cu.

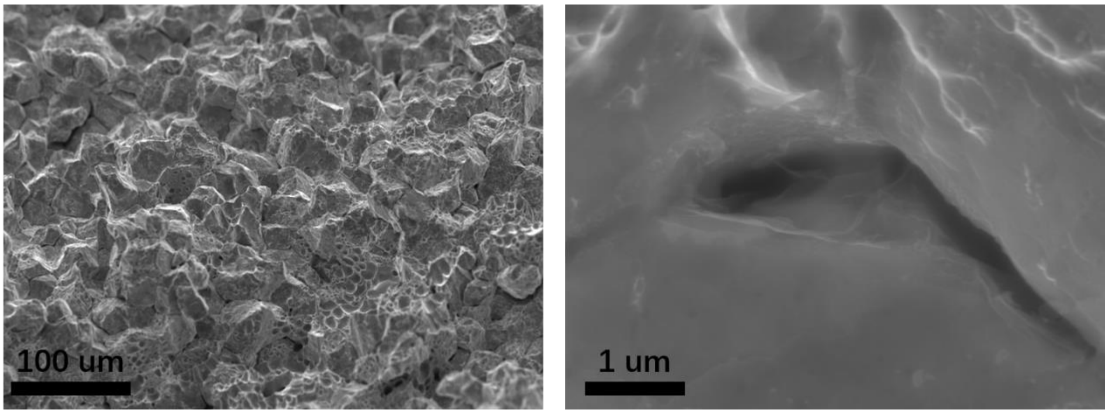

Figure S8. SEM images of fracture surface of the sintered RGO(0.25 wt%)-A-Cu, showing the preserved graphene sheet between the boundaries of particles.

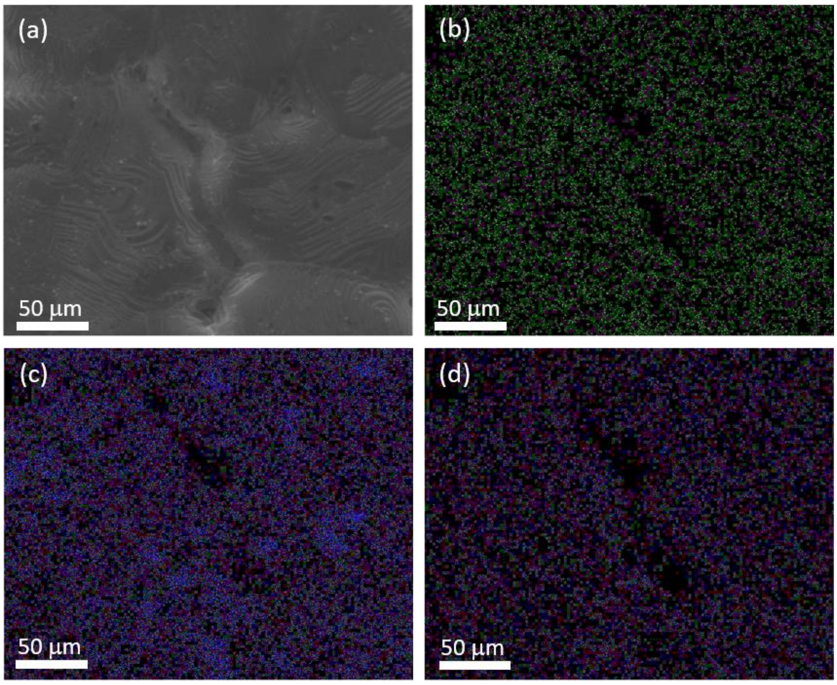

Figure S9. SEM image of (a) sintered GO-Cu and elemental composition maps of (b) Si, (c) O and (d) C.

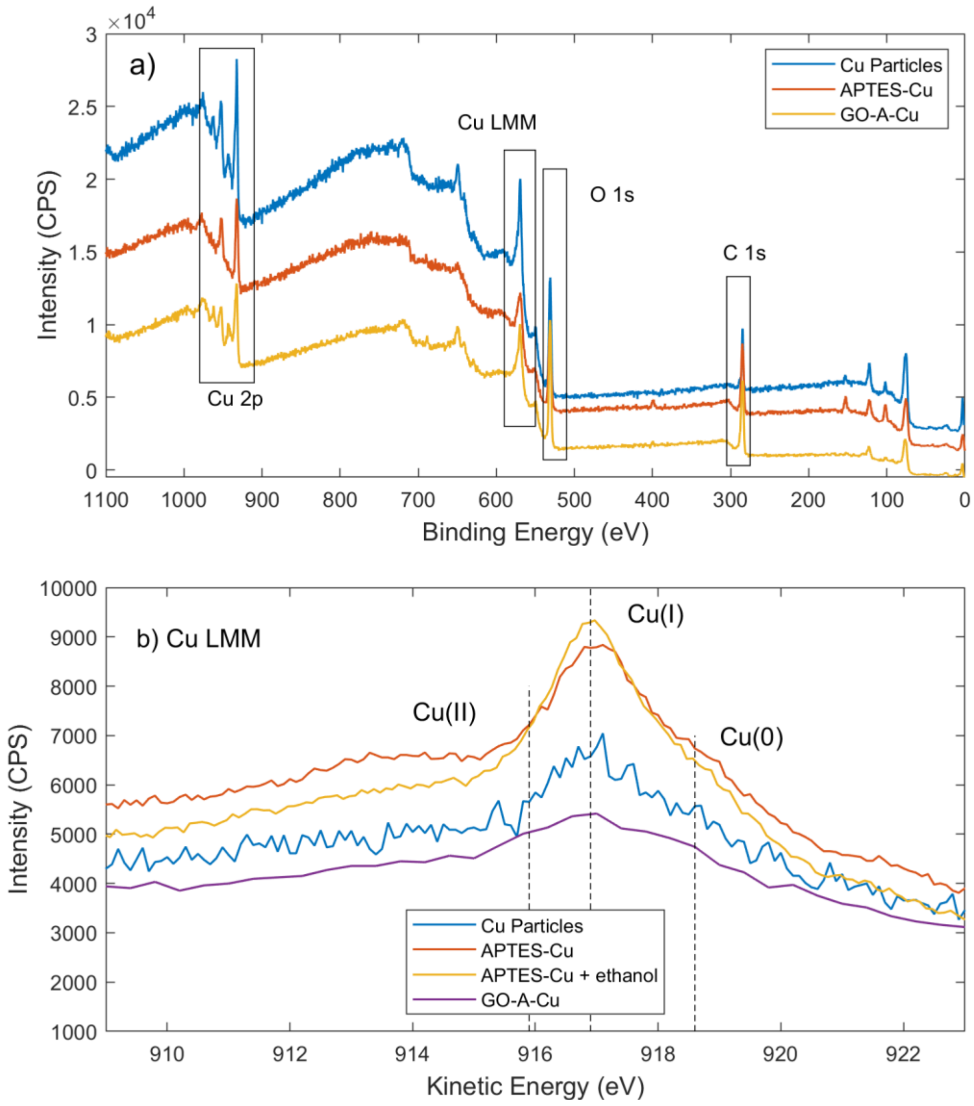

Figure S10. XPS Survey of (a) GO-A-Cu. XPS Cu LMM signal detail (b). Auger modified parameter was within the range 1849.1-1849.3 eV for all samples.

	C	O	Cu_2O	CuO	$Cu(OH)_2$	Si	N
Cu Particles	25	26	39	6.3	4.9	-	-
APTES-Cu	37	31	13.2	3.1	0.2	10.5	4.5
APTES-Cu +ethanol	28	31	35	0.6	1.0	3.0	2.1
GO-A-Cu	56	32	6.2	1.4	1.0	2.2	0.9
RGO-A-Cu	79	16	1	-	-	4.2	-
RGO-A-Cu Sintered	48	35	14	-	-	2.5	-

Table S1. XPS atomic composition (at. %). Errors on C (C 1s) and O (O 1s) are ±1%, errors on Cu (Cu 2p), Si (Si 2p), and N (N 1s) in the range ±0.5/±0.1%

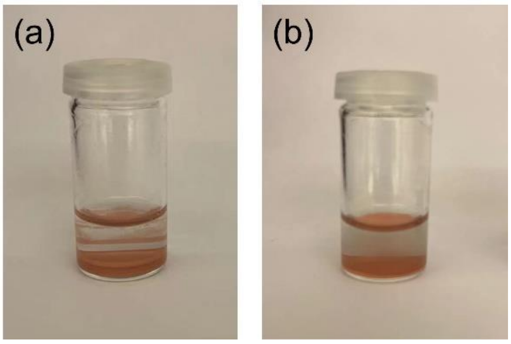

Figure S11. Images of silane coated Cu in 10% acetic acid (a) before and (b) after 19 h.

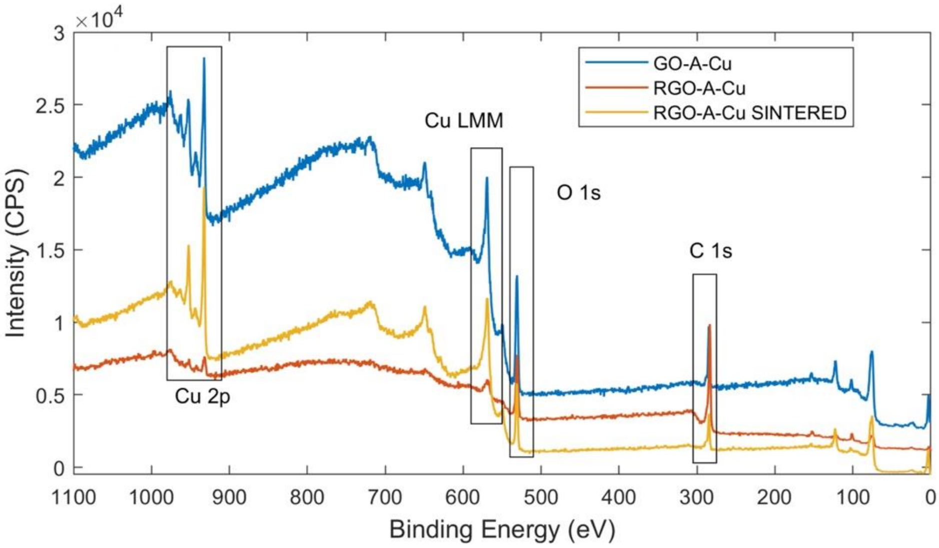

Figure S12. XPS Survey of GO-A-Cu, RGO-A-Cu, and RGO-A-Cu after sintering.

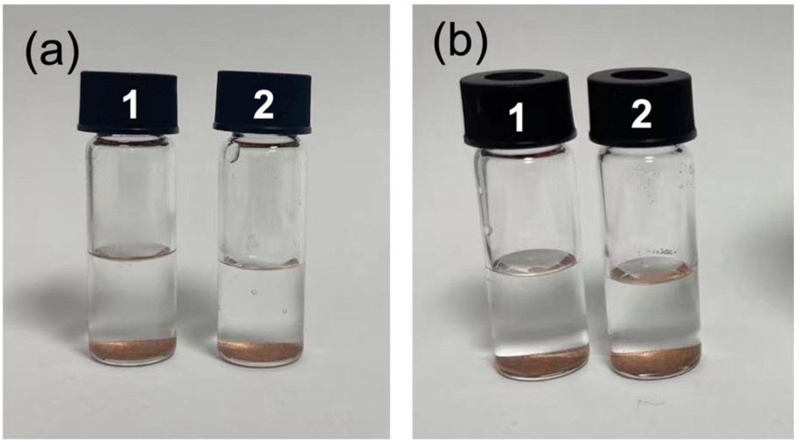

Figure S13. Photos the reduced GO coated Cu (sample 1 without degassing; sample 2

after 10 min degassing by Ar) in 10% acetic acid after 0 h (a) and 24 h (b).

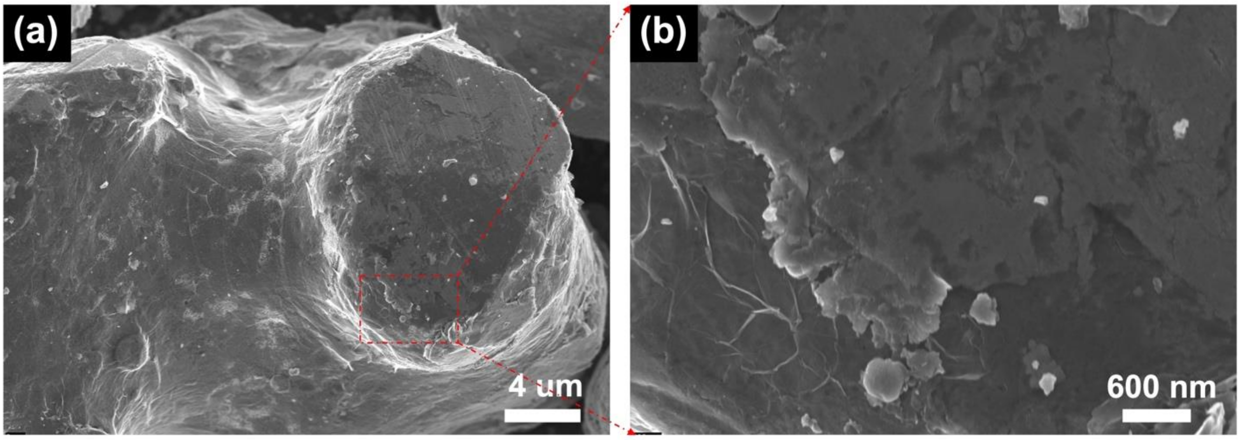

Figure S14. (a and b) The SEM images of RGO-A-Cu particles after grinding; Image b shows the zoom in part of image a.
